# Colorectal Cancer and Long-Term Exposure to Trihalomethanes in Drinking Water: A Multicenter Case–Control Study in Spain and Italy

**DOI:** 10.1289/EHP155

**Published:** 2016-07-06

**Authors:** Cristina M. Villanueva, Esther Gracia-Lavedan, Cristina Bosetti, Elena Righi, Antonio José Molina, Vicente Martín, Elena Boldo, Nuria Aragonés, Beatriz Perez-Gomez, Marina Pollan, Ines Gomez Acebo, Jone M. Altzibar, Ana Jiménez Zabala, Eva Ardanaz, Rosana Peiró, Adonina Tardón, Maria Dolores Chirlaque, Alessandra Tavani, Jerry Polesel, Diego Serraino, Federica Pisa, Gemma Castaño-Vinyals, Ana Espinosa, Nadia Espejo-Herrera, Margarita Palau, Victor Moreno, Carlo La Vecchia, Gabriella Aggazzotti, Mark J Nieuwenhuijsen, Manolis Kogevinas

**Affiliations:** 1 ISGlobal, Centre for Research in Environmental Epidemiology (CREAL), Barcelona, Spain; 2 IMIM (Hospital del Mar Medical Research Institute), Barcelona, Spain; 3 Department of Experimental and Health Sciences, Universitat Pompeu Fabra (UPF), Barcelona, Spain; 4 CIBER Epidemiología y Salud Pública (CIBERESP), Madrid, Spain; 5 Department of Epidemiology, IRCCS (Istituto di Ricerche Farmacologiche Mario Negri), Milan, Italy; 6 Department of Biomedical, Metabolic and Neural Sciences, University of Modena and Reggio Emilia, Modena, Italy; 7 Department of Preventive Medicine and Public Health, University of León, León, Spain; 8 Cancer and Environmental Epidemiology Unit, National Centre for Epidemiology, Carlos III Institute of Health, Madrid, Spain; 9 Cancer Epidemiology Research Group, Oncology and Hematology Area, IIS (Instituto de Investigación Sanitaria) Puerta De Hierro, Madrid, Spain; 10 Department of Preventive Medicine and Public Health, University of Cantabria, Santander, Spain; 11 IDIVAL (Instituto de Investigación Sanitaria Valdecilla), Santander, Spain; 12 Centre for Research in Public Health, Valencia, Spain; 13 Public Health Division of Gipuzkoa, Biodonostia Research Institute, San Sebastian, Spain; 14 Instituto de Salud Pública y Laboral de Navarra, Pamplona, Spain; 15 Oncology Institute IUOPA (Instituto Universitario de Oncología del Principado de Asturias), Universidad de Oviedo, Asturias, Spain; 16 Department of Epidemiology, Murcia Regional Health Council, IMIB-Arrixaca (Biomedical Research Institute of Murcia), Murcia, Spain; 17 Unit of Epidemiology and Biostatistics, CRO (Centro di Riferimento Oncologico) Aviano National Cancer Institute, IRCCS, Aviano, Italy; 18 Institute of Hygiene and Clinical Epidemiology, University Hospital of Udine, Udine, Italy; 19 Department of Biological and Medical Sciences, University of Udine, Udine, Italy; 20 General Division of Public Health, Quality and Innovation, Ministry of Health, Social Services and Equity, Madrid, Spain; 21 Catalan Institute of Oncology, Bellvitge Biomedical Research Institute (IDIBELL), Barcelona, Spain; 22 Department of Clinical Sciences, University of Barcelona, Barcelona, Spain; 23 Department of Clinical Sciences and Community Health, Università degli Studi di Milano, Milan, Italy

## Abstract

**Background:**

Evidence on the association between colorectal cancer and exposure to disinfection by-products in drinking water is inconsistent.

**Objectives:**

We assessed long-term exposure to trihalomethanes (THMs), the most prevalent group of chlorination by-products, to evaluate the association with colorectal cancer.

**Methods:**

A multicenter case–control study was conducted in Spain and Italy in 2008–2013. Hospital-based incident cases and population-based (Spain) and hospital-based (Italy) controls were interviewed to ascertain residential histories, type of water consumed in each residence, frequency and duration of showering/bathing, and major recognized risk factors for colorectal cancer. We estimated adjusted odds ratios (OR) for colorectal cancer in association with quartiles of estimated average lifetime THM concentrations in each participant’s residential tap water (micrograms/liter; from age 18 to 2 years before the interview) and estimated average lifetime THM ingestion from drinking residential tap water (micrograms/day).

**Results:**

We analyzed 2,047 cases and 3,718 controls. Median values (ranges) for average lifetime residential tap water concentrations of total THMs, chloroform, and brominated THMs were 30 (0–174), 17 (0–63), and 9 (0–145) μg/L, respectively. Total THM concentration in residential tap water was not associated with colorectal cancer (OR = 0.92, 95% CI: 0.66, 1.28 for highest vs. lowest quartile), but chloroform concentrations were inversely associated (OR = 0.31, 95% CI: 0.24, 0.41 for highest vs. lowest quartile). Brominated THM concentrations showed a positive association among men in the highest versus the lowest quartile (OR = 1.43, 95% CI: 0.83, 2.46). Patterns of association were similar for estimated average THM ingestion through residential water consumption.

**Conclusions:**

We did not find clear evidence of an association between detailed estimates of lifetime total THM exposure and colorectal cancer in our large case–control study population. Negative associations with chloroform concentrations and ingestion suggest differences among specific THMs, but these findings should be confirmed in other study populations.

**Citation:**

Villanueva CM, Gracia-Lavedan E, Bosetti C, Righi E, Molina AJ, Martín V, Boldo E, Aragonés N, Perez-Gomez B, Pollan M, Gomez Acebo I, Altzibar JM, Jiménez Zabala A, Ardanaz E, Peiró R, Tardón A, Chirlaque MD, Tavani A, Polesel J, Serraino D, Pisa F, Castaño-Vinyals G, Espinosa A, Espejo-Herrera N, Palau M, Moreno V, La Vecchia C, Aggazzotti G, Nieuwenhuijsen MJ, Kogevinas M. 2017. Colorectal cancer and long-term exposure to trihalomethanes in drinking water: a multicenter case–––control study in Spain and Italy. Environ Health Perspect 125:56–65; http://dx.doi.org/10.1289/EHP155

## Introduction

Colorectal cancer represents nearly 10% of global cancer incidence, with increasing rates over the last decades (Bosman et al. 2014). Intake of total energy, red and processed meat, and alcoholic drinks together with physical inactivity, body fat, abdominal fat, and adult-attained height are established risk factors [Bosman et al. 2014; World Cancer Research Fund (WCRF) and American Institute for Cancer Research (AICR) et al. 2011]. Suggested protective factors include consumption of dietary fiber and high fruit and vegetable consumption (WCRF and AICR et al. 2011; Bradbury et al. 2014), among others. However, part of the burden of disease remains unexplained by the above-mentioned risk factors. Human and animal studies suggest that carcinogens in drinking water may be associated with colorectal cancer risk (Rahman et al. 2010).

Disinfection by-products (DBPs) are chemicals resulting from disinfection processes that are widespread in drinking water. Trihalomethanes (THMs) are among the most prevalent DBPs and are highly volatile and skin permeable; exposure occurs through inhalation, dermal absorption, and ingestion (Ashley et al. 2005; Gordon et al. 2006). The four THMs in drinking water that are regulated in the United States, European Union, and other countries are chloroform, bromodichloromethane, dibromochloromethane, and bromoform; these chemicals display different physicochemical and toxicological properties. Chloroform is highly volatile, whereas the other three compounds (from now on referred to as brominated THMs) are more lipophilic and genotoxic (Plewa et al. 2008). Metabolism of DBPs is mediated by enzymes from the cytochrome P450 (CYP) and glutathione *S*-transferase (GST) families. In a hospital-based case–control study, polymorphisms in *CYP2E1*, *GSTT1* and *GSTZ1* were found to modify associations between bladder cancer and DBP exposure (Cantor et al. 2010).

Animal studies have suggested an association between DBP exposure and colorectal cancer. Preneoplastic lesions have been produced in the intestines of rodents administered DBPs via drinking water in chronic bioassays (DeAngelo et al. 2002; McDorman et al. 2003). However, some studies have shown that chloroform may inhibit gastrointestinal carcinogenecity in rodents (Daniel et al. 1989, 1991). Human epidemiological evidence is mixed. Case–control (Bove et al. 2007; Cragle et al. 1985; Hildesheim et al. 1998; King et al. 2000; Young et al. 1987) and cohort (Doyle et al. 1997; Koivusalo et al. 1997) studies including incident cases of colorectal cancer and quantitative estimates of exposure to DBPs such as trihalomethanes or related surrogates have produced contradictory results (see Table S1). Three studies reported positive associations with colon cancer (Cragle et al. 1985; Doyle et al. 1997; King et al. 2000), and three studies reported null associations (Hildesheim et al. 1998; Koivusalo et al. 1997; Young et al. 1987). Similarly, two studies reported positive associations with rectal cancer (Bove et al. 2007; Hildesheim et al. 1998), and three reported null associations (Doyle et al. 1997; King et al. 2000; Koivusalo et al. 1997). The exposure assessments differed, with five studies evaluating THMs (Bove et al. 2007; Doyle et al. 1997; Hildesheim et al. 1998; King et al. 2000; Young et al. 1987) and one each evaluating years living in households with chlorinated water (Cragle et al. 1985) and water mutagenicity (Koivusalo et al. 1997). The inconsistency of human epidemiological evidence might be partly attributable to exposure misclassification, including a lack of evaluation of different routes of exposure and uncontrolled confounders.

Here, we examined the association between THM exposure and colorectal cancer in a large multicenter case–control study considering specific THM components and exposure activities involving dermal contact, inhalation, and ingestion.

## Methods

### Study Design and Population

***Spain.*** We conducted a multicase–control study from September 2007 to November 2013 in nine provinces of Spain (MCC-Spain project) including colorectal, prostate, breast, and gastroesophageal cancer and chronic lymphocytic leukemia cases and a common pool of population-based controls (Castaño-Vinyals et al. 2015). Cases included in the present analysis were histologically confirmed incident colorectal cancer patients [*International Statistical Classification of Diseases and Related Health Problems* (10th revision) (ICD-10): C18, C19, C20, D01.0, D01.1, D01.2]. Cases were interviewed as soon as possible after diagnosis (median of 58 days) and were identified through active searches including periodic visits to hospital departments (i.e., gastroenterology, oncology, general surgery, radiotherapy, and pathology). Minimal losses occurred from cases dying before being contacted (0.5% of potentially eligible cases). Controls were frequency matched to cases by sex, age (± 5 years), and area of residence, ensuring that in each area there was at least one control of the same sex and 5-year interval for each case. Controls were selected from the general population in each study area and were identified from the lists of randomly selected family practitioners in primary assistance centers sharing the catchment area of the participating hospital. This procedure ensured the identification of controls from the general population and from the same study base as cases, through the social security records (health coverage is universal in Spain), with the advantage that the telephone contacts by the study personnel on behalf of the family practitioner increased the response rate compared with other procedures (Castaño-Vinyals et al. 2011). For each control needed, five potential participants of similar age, sex, and hospital catchment area were randomly selected from the general practitioner lists. If contact was not made with the first person on the list after five tries at different times of day, or if he/she refused to participate, the next person on the list was approached (Castaño-Vinyals et al. 2011). The frequency matching was done separately for each study area, considering the age and sex distribution of the total number of cases recruited. Thus, the final number of controls in the present analysis is greater than the number of cases because the controls were matched to different cancer sites.

***Italy.*** We conducted a case–control study of colorectal cancer in three provinces of Italy (Milan, Pordenone, Udine) between January 2008 and July 2010 (Hiwate Project) (Nieuwenhuijsen et al. 2009). Cases were histologically confirmed incident colorectal cancer patients (ICD-10: C18, C19, C20) who were interviewed as soon as possible after diagnosis (median of 28 days) and were identified through active searches including periodic visits to hospital departments (i.e., gastroenterology, oncology, general surgery, radiotherapy, and pathology). Controls were frequency matched to cases by sex, age (± 5 years), and area of residence, with a case:control ratio of 1. Controls were identified from patients admitted to the same network of hospitals as cases for a wide spectrum of acute, non-neoplastic conditions unrelated to tobacco and alcohol consumption, long-term diet modification, and other factors likely related to colorectal cancer. Overall, 52% of controls had acute surgical conditions, 15% had orthopedic disorders, 12% had dental, ear, nose and throat diseases, and 21% had miscellaneous other conditions.

In both countries, study participants were 20–85 years old, had resided in the hospital catchment area for ≥ 6 months before recruitment, and were able to answer the epidemiological questionnaire. Participant hospitals (17 in Spain, 10 in Italy) were the reference centers for oncologic diseases in each study area. The study protocol was approved by the ethics review boards of the participating centers, and all participants signed an informed consent before recruitment.

### Data Collection and Response Rates

Trained interviewers administered questionnaires to collect personal information on sociodemographic factors; lifestyle (smoking, alcohol consumption, physical activity, etc.); anthropometrics (height, weight); residential history; type of water consumed in each residence (municipal, bottled, other); frequency of showering, bathing, and hand dishwashing; occupation; medication; medical history; and family history of cancer. Amount of water ingested was ascertained as the number of glasses per day consumed on average by an adult at home, at the workplace and in other places; bottled water, municipal water, water from other sources, coffee, tea, and herbal drinks were considered separately. A final section evaluating the quality of the interview was completed by the interviewer. Dietary habits were collected through semiquantitative food frequency questionnaires that were self-administered in Spain and administered by an interviewer in Italy. Sample size was fixed by the primary scientific objectives of the MCC-Spain Study (Castaño-Vinyals et al. 2015). Before any analysis, we used data from the literature on total THM distribution (Villanueva et al. 2012) to calculate [using the program GRANMO 7.12 (Marrugat et al. 1998)] that the statistical power was 100%, assuming a difference of 4 μg/L between cases and controls (based on current preliminary data), and accepting an alpha risk of 0.05 in a two-sided contrast. Response rates were calculated as follows: subjects interviewed were in the numerator, and all subjects including refusals were in the denominator. The average response rates were 68% in Spain and 95% in Italy among cases, and 53% in Spain and 95% in Italy among controls. In total, 2,371 cases (1,905 in Spain, 466 in Italy) and 4,159 controls (3,590 in Spain, 569 in Italy) were interviewed.

### Trihalomethane Levels in the Study Areas

We used trihalomethanes as a surrogate of DBPs. We collected routine THM measurements (chloroform, bromodichloromethane, dibromochloromethane, and bromoform) and historical information on water sources (surface/ground proportion) and treatment in the drinking water supplied to the study areas back to 1940 through a structured questionnaire aimed at water utilities and local and health authorities. Routine monitoring data were provided by the SINAC (Sistema de Información Nacional en Aguas de Consumo) in Spain (2004–2010) and by the Regional Environmental Health Agency (Milan) and the Local Health Authority (Pordenone/Udine) in Italy. Availability of THM measurements differed among study areas, with the longest record starting in 1979. In total, 275 water zones in Spain and 370 in Italy were included.

### Estimation of Long-Term Trihalomethane Levels in Drinking Water

Annual average levels were calculated for the four THMs separately at the water zone level, that is to say, at the minimum geographic unit with homogeneous water source, treatment, and quality (corresponding to municipality in most cases). Cases and controls lived on average in three residences during the exposure window; therefore, cases and controls from an area had different and multiple water zones assigned. Years without measurements were assigned the average of all available measurements in the water zone if the water source and treatment did not change over the years. Trihalomethane concentrations in surface water are usually higher than in ground sources (Villanueva et al. 2003), and we used surface water percentage as a weight to back-extrapolate THM concentrations when water sources changed, assuming that concentrations increased proportionately to the percentage of surface water. The same assumptions were used for the four THMs and were applied uniformly in all water zones. Hypochlorite was the main disinfectant used in all of the study areas. Alternative or complementary treatments identified in the study areas included permanganate, chlorine dioxide, and ozonation. Water zones with THM measurements and changes in treatment over the years were used to estimate the change percentage in the four THMs separately after introducing such treatments. These percentages were applied as a weight to back-extrapolate concentrations of the four THMs separately in areas with changes in these specific treatments for years when measurements were unavailable. Before chlorination started, THM concentrations were assumed to be zero. The years when chlorination started varied widely among study areas, ranging from before 1940 (Asturias, Barcelona, Cantabria) to 2005 (a small municipality in Valencia). Using these procedures, we estimated annual average THM levels by water zone back to 1940 in areas covering 83% of study person-years, ranging from 67% (León) to 95% (Gipuzkoa).

### Individual Exposures in the Study Population

***Average THM concentrations in residential tap water.*** Trihalomethane concentrations (micrograms/liter; considering the four THMs separately) and subjects’ personal data were linked by year and water zone of residence to obtain annual concentrations in the residences where subjects lived from age 18 to 2 years before the interview (henceforth referred to as “lifetime”). The average concentration in all residences with THM estimates was then calculated.

***Average THM ingestion.*** The average THM ingestion (micrograms/day) was estimated by calculating the average residential THM concentration assuming a zero level in residences consuming bottled water [based on a report by Font-Ribera et al. (2010)] and 0.3, 0.3, 0.8, and 1.8 μg/L for chloroforom, bromodichloromethane, dibromochloromethane, and bromoform, respectively, in residences consuming private well water. These well-water values were averages of 56 observations from water zones with chlorinated ground water in different areas (Gipuzkoa, Barcelona, Valencia, and Navarra) because we assumed that private well water was likely to be chlorinated. Municipal THM concentration was assigned to the years living in residences with municipal water consumption. We averaged the THM concentration calculated using this procedure in residences from age 18 to 2 years before the interview and multiplied by the amount of water consumed at home (liters/day, assuming 200 mL/glass). We used total water consumed at the residence (municipal, bottled, private well) because we did not have the specific amount by residence, and these habits may have changed over a participant’s life.

***Showering and bathing THM exposure.*** We evaluated THM exposure through showering and bathing (micrograms/liter × minutes/day) in terms of the average duration of showers and baths (minutes/week) and the average THM concentration in residential tap water.

### Genotyping

Blood or saliva samples were collected in all Spanish areas for DNA analysis. The response rate among those included in the present analysis was 93%, and DNA was obtained from 3,579 blood and 926 saliva samples. Owing to budget restrictions, genotyping was performed in a random sample of subjects (except in the Murcia area, where genotyping was not conducted) covering 71% of the Spanish participants. Genome-wide genotyping was performed using a customized version of the Infinium Human Exome BeadChip Kit v.1.1 (Illumina). A total of 5,363 single nucleotide polymorphisms (SNPs) of interest to the MCC study were added to the chip, for a total of 248,264 markers. The genotype calling was performed using the GenTrain 2.0 algorithm (GenomeStudio software) based on CHARGE clusters (Grove et al. 2013). PLINK was used for the genetic data quality control (Purcell et al. 2007), and standard checks were performed: sample call rate, sex concordance, heterozygosity, relatedness, population stratification, minimum SNP call rate of 95%, and departure from Hardy–Weinberg equilibrium (HWE) for each SNP tested among Spanish controls. After quality control, the final database included 232,396 SNPs, with 94,659 monomorphic and 99,411 with a minor allele frequency (MAF) < 1%, 8,853 with MAF 1–5%, and 29,473 with MAF > 5%. For the present analysis, we selected SNPs in *CYP2E1* and *GSTZ1* genes based on prior work by Cantor et al. (2010). After genotyping quality controls, 22 SNPs in *CYP2E1* and 7 SNPs in *GSTZ1* were available for statistical analysis (see Table S2 for a complete list). Genetic data were available for 981 cases and 2,204 controls, which corresponds to a random sample of participants from Spain.

### Covariables

Smokers were defined as those smoking at least 100 cigarettes or 360 g of tobacco in life on a regular basis, that is, at least one cigarette/day for ≥ 6 months. Former smokers were defined as those who quit smoking ≥ 1 year before the interview. Users of nonsteroidal antiinflammatory drugs (NSAIDs) were coded as ever (or never) users if subjects consumed (or not) NSAIDs at least 30 times over life. Physical activity was ascertained through open questions on any type of physical activity practiced in life, the years and the frequency (hours/week) converted to metabolic equivalents (METs) from age 16 (Spain) or 15 (Italy) to 2 years before the interview. Cancer diagnosis (any type) in first-degree relatives was asked, and for positive responses, the type of cancer was ascertained. Family history of colorectal cancer was defined by self-reported malignant tumors (polyps excluded). Body mass index was calculated based on the weight 1 year before the interview and was categorized in three groups (< 25, 25–29.9, ≥ 30 kg/m^2^). A total of 156 food items were ascertained through a food frequency questionnaire, assessing usual dietary intake the year before the interview. Frequencies in servings/day of red meat, processed meat, dairy products, fruits, and vegetables were converted to grams/day based on food composition tables.

### Statistical Analysis

Only subjects with average THM concentrations in the residential tap water, average THM ingestion, and average showering/bathing THM exposure estimates for ≥ 70% of the years between age 18 and 2 years before the interview (89% of interviewed subjects) were considered in the respective models, and subjects with unreliable interviews (*n* = 16) were excluded. Thus, a total of 5,765 subjects were included in the statistical analyses (2,047 cases, 3,718 controls), of which 5,291 (1,837 cases, 3,454 controls) were analyzed for average THM concentrations in residential tap water and 5,731 (2,047 cases, 3,684 controls) were analyzed for ingested THMs. The higher numbers for the ingestion variable are explained by those whose residential THM concentration cannot be estimated and who consumed bottled or private well water. We used unconditional logistic regression to estimate odds ratios (ORs) and 95% confidence intervals (CIs) for associations between colorectal cancer and THM exposures adjusted for age (continuous), sex, area (as a factor in the model), education, smoking, use of NSAIDs, leisure physical activity, and family history of colorectal cancer (first-degree relatives), categorized as shown in Table 1. Missing values in categorical variables were coded as a separate category. There were no missing values for the matching variables (age, sex, area). Categories of body mass index and milligrams/day (continuous) of red meat, processed meat, dairy product, fruit, and vegetable consumption were explored but did not meet our criterion for confounding (< 10% change in the risk estimates) and were omitted to keep the most parsimonious models. Given the different genotoxicity (Landi et al. 1999) and collinearity among THMs, analyses were conducted separately for chloroform and the brominated THMs (bromodichloromethane, dibromochloromethane, and bromoform), and mutually adjusted ORs for both exposures were not estimated. Exposure categories for both cases and controls were created according to quartiles of THM exposure overall. Linear *p*-trends were calculated using a likelihood-ratio test comparing the model with and without the exposure variable with numerically coded quartiles (0, 1, 2, 3). Generalized additive models (GAMs) were used to evaluate the exposure–response relationships of continuous variables, using a smoothed spline with 3 degrees of freedom with the *gam* function in Stata release 12 (StataCorp LP). Departure from linearity was assessed by testing the difference in normalized deviance between the GAM and a model with a linear term for the exposure, based on a chi-squared approximation. Other exposure variables examined included the type of water consumed at the place of longest residence and showering/bathing frequency combined with THM concentrations (minutes/day × micrograms/liter). The main analyses were stratified by sex, given the higher incidence among men, expected differences in established risk factors, and for comparability with previous studies. We explored potential differences by cancer site (colon, rectum) in stratified analyses for comparability with previous studies. The *p*-value threshold defining statistical significance was set at < 0.05. Interactions between lifetime average THM concentrations at the residence (dichotomized at the 75th percentile) and genotypes were examined to assess the odds ratios within genotype categories; the dominant model (heterozygous and homozygous variant versus homozygous wild type) was used. We used the likelihood-ratio test to evaluate the joint effects of SNPs and the interaction term. Linkage disequilibrium was evaluated, and the number of effective tests with an *R* ^2^ cut-off set at 0.8 was 15. Bonferroni corrections for number of effective tests were applied in genotype analyses.

**Table 1:** Characteristics of the study population: 2,047 cases of colorectal cancer and 3,718 controls.

Variable	Cases *n* (%) or mean ± SD	Controls *n* (%) or mean ± SD
Age (years), mean (SD)	67 ± 10	64 ± 11
Sex
Men	1,317 (64.3)	1,943 (52.3)
Women	730 (35.7)	1,775 (47.7)
Area
Asturias (Spain)	70 (3.4)	211 (5.7)
Barcelona (Spain)	635 (31.0)	957 (25.7)
Cantabria (Spain)	125 (6.1)	282 (7.6)
Guipuzcoa (Spain)	113 (5.5)	329 (8.9)
León (Spain)	294 (14.4)	360 (9.7)
Madrid (Spain)	204 (10.0)	670 (18.0)
Murcia (Spain)	28 (1.4)	39 (1.1)
Navarra (Spain)	102 (5.0)	234 (6.3)
Valencia (Spain)	76 (3.7)	133 (3.6)
Milano (Italy)	215 (10.5)	301 (8.1)
Pordenone/Udine (Italy)	185 (9.0)	202 (5.4)
Education
< Primary school	519 (25.4)	592 (15.9)
Primary school	766 (37.5)	1,234 (33.2)
Secondary school	566 (27.7)	1,182 (31.8)
University	190 (9.3)	707 (19.0)
Missing	6	3
Smoking status
Never	841 (41.4)	1,629 (43.9)
Former	821 (40.4)	1,290 (34.8)
Current	371 (18.2)	789 (21.3)
Missing	14	10
Nonsteroidal antiinflammatory drug consumption^*a*^
No	1,382 (69.7)	2,237 (62.3)
Yes	600 (30.3)	1,352 (37.7)
Missing	65	129
Body mass index (kg/m^2^)^*b*^
< 25	676 (33.0)	1,391 (37.8)
25–29.9	909 (44.4)	1,555 (42.3)
≥ 30	461 (22.5)	734 (20.0)
Missing	1	38
Leisure physical activity (adult life average)^*c*^
0 METs	627 (30.6)	1,011 (27.4)
0–8 (8.5) METs	698 (34.1)	1,324 (35.9)
8–16 (34.4) METs	294 (14.4)	582 (15.8)
> 16 (34.4) METs	428 (20.9)	767 (20.8)
Missing	0	34
Family history of colorectal cancer (first degree)
No	1,648 (85.4)	3,246 (92.0)
Yes	281 (15.6)	284 (8.0)
Missing	118	188
Drinking-water type at the place of longest residence
Tap (municipal)	1,185 (58%)	2,474 (67%)
Bottled	655 (32%)	1,014 (27%)
Private well	198 (10%)	227 (6%)
Missing	9	3
Number of residences	3.2 ± 1.5	3.3 ± 1.6
Years at the place of longest residence	35.6 ± 12.0	31.8 ± 12.1
Average THM concentration in residential tap water (μg/L)^*d*^	44.8 ± 43.9	40.3 ± 34.2
Average THM ingestion from residential tap water (μg/day)	28.1 ± 48.8	25.9 ± 36.4
Average showering/bathing THM concentrations (min/day × μg/L)^*e*^	284.7 ± 406.4	266.6 ± 311.5
Notes: MET, metabolic equivalent; THM, trihalomethane. The table excludes subjects with inadequate THM exposure data and 16 observations with low-quality interviews. ^***a***^Ever users consumed nonsteroidal antiinflammatory drugs ≥ 30 times in life. ^***b***^Body mass index based on the weight 1 year before the interview. ^***c***^Categories were country-specific, and cut-offs for Italy are in parentheses. ^***d***^Number of observations: 1,977 cases, 3,679 controls. ^***e***^Number of observations: 1,834 cases, 3,480 controls.		

## Results

A total of 5,765 subjects were included in the present analysis: 1,647 cases and 3,215 controls from Spain, and 400 cases and 503 controls from Italy (Table 1). The area contributing with the largest population was Barcelona (28% of all subjects), followed by Madrid (15%). The proportion of men was higher among cases (64%) than controls (52%). Cancer location was colon for 1,410 (69%) cases, rectum for 607 (30%) cases, and unspecified or missing for 30 (1%) cases. The proportions of smokers, participants with BMI > 25 kg/m^2^, and family history of colorectal cancer was higher among cases, whereas the proportion of NSAID users was higher among controls, and physical activity was similar among cases and controls (Table 1). Water type consumed in the place of longest residence, residential history, and THM exposure are presented in Table 1.

**Figure 1:**
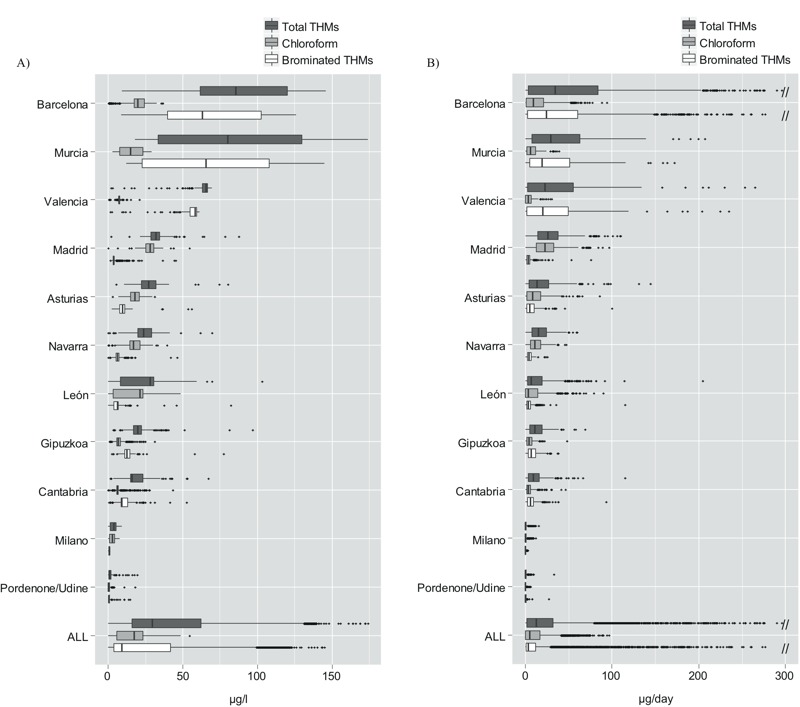
Distribution of average residential trihalomethane (THM) exposure among subjects with THM estimates for ≥ 70% of the exposure window (age 18 to 2 years before the interview) from reliable interviews. (*A*) Average concentrations at the tap (micrograms/liter), *n* = 5,291. (*B*) Average ingested levels (micrograms/day), *n* = 5,731. All areas correspond to Spain except Milano and Pordenone/Udine (Italy). Boxes are delimited by the 25th (left hinge) and 75th (right hinge) percentiles, the central vertical line represents the median value, the whiskers represent ± 1.5 times the interquartile range, and the points outside the whiskers represent outliers.

The concentration and composition of trihalomethanes differed between study areas (Figure 1). The median concentrations overall were 29.5, 17.4, and 9.0 μg/L for residential total THMs, chloroform, and brominated THMs, respectively. Area median concentration of total THMs ranged from 1 μg/L (Udine/Pordone, Italy) to 86 μg/L (Barcelona). Brominated THMs were highest in Murcia and Barcelona, with medians of 65 and 64 μg/L, respectively, and chloroform was highest in Madrid (median 28 μg/L). Differences in ingested THMs among areas were less pronounced than concentrations of THMs in drinking water because the areas with higher THM concentrations usually had higher proportions of bottled water consumption. The Spearman correlation coefficients between total THMs versus chloroform, bromodichloromethane, dibromochloromethane, and bromoform were 0.68, 0.87, 0.77, and 0.64, respectively. Between total brominated THMs and bromodichloromethane, dibromochloromethane and bromoform, the Spearman correlation coefficients were 0.96, 0.98, and 0.90, respectively. These correlations varied among study areas. Total THM–chloroform correlation was > 0.84 in all areas except Barcelona (–0.32). The distribution densities of average lifetime THM concentrations in residential tap water by case–control status and by area are shown in Figure S1.

**Figure 2:**
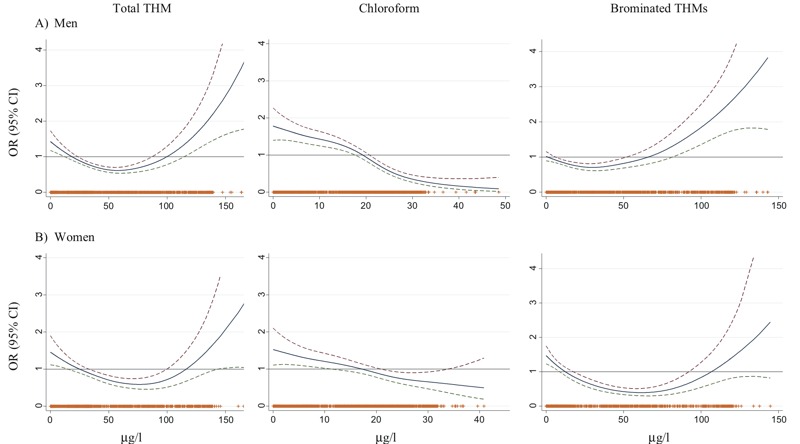
Exposure–response relationship between residential trihalomethane (THM) concentrations (*x*-axis, in micrograms/liter) and colorectal cancer (*y*-axis, expressed as odds ratios, ORs) among 1,837 cases and 3,454 controls. (*A*) Men. (*B*) Women. Smoothed spline with 3 degrees of freedom from general additive models adjusted for sex, age, area, education, smoking, physical activity, nonsteroidal antiinflammatory drug use, and family history of cancer. Subjects with unsatisfactory questionnaires and subjects with THMs estimated for < 70% of the exposure window were excluded. *p*-Value gain compared with linearity is < 0.001 for all models, except for chloroform in women (*p*-value = 0.38). Tick marks above the *x*-axes represent observations, and the dashed lines represent the 95% confidence intervals (CIs).

Lifetime average total THM concentrations were not positively associated with colorectal cancer among men or women, and a negative association was observed for some exposure categories in the low-exposure range (Table 2). Chloroform concentrations were negatively associated with colorectal cancer, with the strongest associations for the highest versus lowest quartile both among men and women. Brominated THM concentrations were positively associated with colorectal cancer among men in the highest versus lowest exposure category (OR = 1.43, 95% CI: 0.83, 2.46) and negatively associated in the third versus lowest quartile (OR = 0.57, 95% CI: 0.36 0.90) and among women (Table 2). Generalized additive models confirmed these patterns, revealing nonlinear exposure–response relationships (Figure 2). However, the curves have low precision at the high end of the exposure ranges, and the estimates are based on small numbers of subjects. Exposure–response curves for the four THMs, for men and women combined, are shown in Figure S2. Approximately linear negative associations are shown for chloroform and positive associations are shown for bromoform, whereas the associations for bromodichloromethane and dibromochloromethane are nonlinear. Associations by area were heterogeneous, with Barcelona showing similar results to the overall estimates (see Figures S3 and S4). Conversely, models with all areas except Barcelona show attenuated dose–response relationships (see Figures S3 and S4), indicating that Barcelona, with ~26% of all controls, ~31% of all cases, and observations in the highest quartiles of brominated THMs, drives the overall results.

**Table 2:** Association between average trihalomethane (THM) concentrations in residential tap water and colorectal cancer. Odds ratios (ORs) and 95% confidence intervals (CIs) among 1,837 cases and 3,454 controls.

Exposure	All (*n* = 5,291)			Men (*n* = 2,977)			Women (*n* = 2,312)^*a*^		
	Cases	Controls	OR^*b*^ (95% CI)	Cases	Controls	OR^*b*^ (95% CI)	Cases	Controls	OR^*b*^ (95% CI)
Total THMs (μg/L)
< 16.2	591	741	1	393	447	1	196	294	1
16.2–29.5	378	954	0.57 (0.45, 0.72)	241	407	0.56 (0.41, 0.77)	137	547	0.62 (0.41, 0.93)
29.5–62	323	1,007	0.44 (0.33, 0.58)	193	530	0.38 (0.27, 0.55)	130	477	0.60 (0.38, 0.93)
> 62	545	752	0.92 (0.66, 1.28)	349	417	1.04 (0.68, 1.60)	196	335	0.68 (0.39, 1.20)
*p-Trend*^*c*^			*0.187*			*0.741*			*0.165*
Chloroform (μg/L)
< 6	589	744	1	387	446	1	200	298	1
6–17.4	435	895	0.69 (0.55, 0.87)	268	404	0.69 (0.51, 0.93)	167	491	0.71 (0.49, 1.03)
17.4–23.4	515	798	0.68 (0.53, 0.87)	335	384	0.73 (0.53, 1.01)	180	414	0.65 (0.43, 0.97)
> 23.4	298	1,017	0.31 (0.24, 0.41)	186	567	0.26 (0.18, 0.37)	112	450	0.46 (0.29, 0.72)
*p-Trend*^*c*^			*< 0.001*			*< 0.001*			*0.001*
Brominated THMs (μg/L)
< 3.7	541	792	1	348	483	1	191	309	1
3.7–9.0	387	943	0.77 (0.60, 0.98)	238	419	0.73 (0.53, 1.01)	149	524	0.78 (0.52, 1.17)
9.0–41.8	368	963	0.55 (0.38, 0.79)	241	479	0.57 (0.36, 0.90)	127	484	0.49 (0.26, 0.91)
> 41.8	541	756	1.00 (0.65, 1.53)	349	420	1.43 (0.83, 2.46)	192	336	0.44 (0.21, 0.92)
*p-Trend*^*c*^			*0.556*			*0.036*			*0.039*
^***a***^Two female cases were excluded from the sex-stratified model because one category in education does not have observations among controls and was dropped from the model. ^***b***^Adjusted for sex, age, area, education, nonsteroidal antiinflammatory drug consumption, smoking, physical activity, and family history of colorectal cancer. The analysis excludes subjects with poor or questionable interviews and with known THM concentrations for < 70% of the exposure window (from age 18 to 2 years before the interview). ^***c***^Linear trend *p*-value, derived from a likelihood-ratio test comparing a model with the categorical nitrate variable as an ordinal variable (0, 1, 2), with a model that excluded the variable.									

Interactions between exposure to total and brominated THMs and 22 selected polymorphisms were evaluated. Nominal statistically significant interactions were observed between four SNPs in the *CYP2E1* gene (rs2070675, rs915907, rs8192775, and rs743535) in relation to colorectal cancer and exposure to brominated THMs at concentrations > 40 μg/L (Table 3); however, none was statistically significant according to the Bonferroni-corrected *p*-value (0.03). Owing to changes in the reference category and in the sample with genetic data, the ORs for the highest quartile versus quartiles 1–3 combined are ~ 2–4 for brominated and total THMs (Table 3). The results for all SNPs analyzed in relation to different exposure cut-offs for total, chlorinated, and brominated THMs are shown in Table S2.

**Table 3:** Interaction between polymorphisms in *CYP2E1* and trihalomethane (THM) exposure, dichotomized at the 75th percentile. Odds ratios (ORs) and 95% confidence intervals (CIs) among 981 cases and 2,204 controls.

Gene, SNP^*a*^	Genotype	Cases	Controls	Total THMs (> vs. ≤ 60 μg/L)		Brominated THMs (> vs. ≤ 40 μg/L)		Chloroform (> vs. ≤ 20 μg/L)	
				OR (95% CI)^*b*^	Nominal *p*‑value^*c*^	OR (95% CI)^*b*^	Nominal *p*‑value^*c*^	OR (95% CI)^*b*^	Nominal *p*‑value^*c*^
rs2070675	CC	682	1,496	3.5 (2.4, 5.1)	0.029	4.1 (2.7, 6.0)	0.009	0.4 (0.3, 0.6)	0.646
	CT/TT	298	707	2.3 (1.3, 4.0)		2.1 (1.2, 3.7)		0.6 (0.4, 0.9)	
rs915907	CC	710	1,566	3.3 (2.2, 4.7)	0.031	3.9 (2.7, 5.8)	0.009	0.4 (0.3, 0.5)	0.392
	AC/AA	252	618	2.6 (1.4, 5.0)		2.3 (1.2, 4.3)		0.8 (0.5, 1.2)
rs8192775	GG	850	1,884	3.1 (2.2, 4.4)	0.103	3.7 (2.6, 5.3)	0.021	0.5 (0.4, 0.6)	0.383
	AG/AA	131	320	2.7 (1.1, 6.5)		1.7 (0.8, 3.9)		0.5 (0.2, 1.0)
rs743535	GG	838	1,823	3.0 (2.1, 4.3)	0.056	3.7 (2.6, 5.3)	0.019	0.5 (0.3, 0.6)	0.066
	AG/AA	142	381	3.4 (1.5, 7.8)		2.0 (0.9, 4.3)		0.5 (0.3, 0.9)
Notes: SNP, single nucleotide polymorphism. ^***a***^Results for all SNPs are reported in Table S2. ^***b***^Adjusted for sex, age, geographical area and education. ^***c***^Likelihood-ratio test for the joint effect of the SNP and interaction term. Critical *p*-value with Bonferroni corrections, *p *< 0.003.									

The use of bottled water versus municipal water at the place of longest residence (33 years long on average) was positively associated with colorectal cancer among men and women combined [OR = 1.19 (95% CI: 1.04, 1.37)], among men [OR = 1.17 (95% CI: 0.98, 1.39)], and among women [OR = 1.20 (95% CI: 0.96, 1.50)]. Ingested chloroform level was negatively associated with colorectal cancer among men and women (Table 4). Ingested brominated THMs led negative associations among women and among men in quartiles 2 and 3 versus quartile 1, whereas a positive association was observed among men in quartile 4 versus quartile 1 (Table 4). Generalized additive models confirmed these patterns (see Figure S5), although the curves at the high end of the exposure range have low precision, and the estimates are based on small numbers of subjects.

**Table 4:** Association between average trihalomethane (THM) ingestion in the residence and colorectal cancer. Odds ratio (ORs) and 95% confidence intervals (CIs) among 2,047 cases and 3,684 controls.

Exposure	All (*n* = 5,731)			Men (*n* = 3,239)			Women (*n* = 2,490)^*a*^		
	Cases	Controls	OR^*b*^ (95% CI)	Cases	Controls	OR^*b*^ (95% CI)	Cases	Controls	OR^*b*^ (95% CI)
Total THMs (μg/day)
< 1.3	601	836	1	390	491	1	209	345	1
1.3–12.2	555	882	0.98 (0.83, 1.17)	372	448	0.99 (0.79, 1.25)	183	434	1.04 (0.78, 1.39)
12.2–32.3	395	1,039	0.74 (0.61, 0.91)	245	474	0.74 (0.58, 0.96)	150	565	0.83 (0.60, 1.14)
> 32.3	496	927	0.83 (0.68, 1.00)	310	509	0.82 (0.64, 1.05)	186	418	0.86 (0.63, 1.17)
*p-Trend*^*c*^			*0.008*			*0.034*			*0.176*
Chloroform (μg/day)
< 0.2	621	816	1	403	471	1	216	345	1
0.2–5.3	537	897	0.87 (0.74, 1.03)	356	489	0.86 (0.70, 1.05)	181	408	0.98 (0.75, 1.29)
5.3–16.6	477	955	0.82 (0.69, 0.98)	310	435	0.89 (0.71, 1.12)	167	520	0.78 (0.58, 1.04)
> 16.6	412	1,016	0.67 (0.56, 0.82)	248	527	0.63 (0.49, 0.81)	164	489	0.74 (0.54, 1.01)
*p-Trend*^*c*^			*< 0.001*			*0.001*			*0.031*
Brominated THMs (μg/day)
< 0.7	590	847	1	384	496	1	204	351	1
0.7–3.7	521	918	1.04 (0.86, 1.26)	332	467	0.99 (0.78, 1.26)	189	451	1.26 (0.91, 1.74)
3.7–11.9	412	1,024	0.79 (0.65, 0.96)	262	460	0.76 (0.59, 0.98)	150	564	0.85 (0.61, 1.19)
> 11.9	524	895	0.83 (0.68, 1.02)	339	499	0.88 (0.68, 1.14)	185	396	0.83 (0.60, 1.15)
*p-Trend*^*c*^			*0.007*			*0.098*			*0.059*
^***a***^Two female cases were excluded from the sex-stratified model because one category in education did not have observations among controls and was dropped from the model. ^***b***^Adjusted for sex, age, area, education, smoking, physical activity, nonsteroidal antiinflammatory drug consumption, and family history of cancer. Models excluded subjects with poor or questionable questionnaires and with THM ingestion estimated for < 70% of the exposure window (from age 18 to 2 years before the interview). ^***c***^Linear trend *p*-value, derived from a likelihood-ratio test comparing a model with the categorical nitrate variable as an ordinal variable (0, 1, 2) with a model that excluded the variable.									

When shower/bath duration was combined with THM concentrations, positive associations were observed for the highest exposure to brominated THMs among men and null to protective associations were observed for chloroform (Table 5; see also Figure S6).

**Table 5:** Association between colorectal cancer and shower/bath trihalomethane (THM) levels. Odds ratios (ORs) and 95% confidence intervals (CIs) among 1,702 cases and 3,269 controls.

Exposure	All (*n* = 4,971)			Men (*n* = 2,793)			Women (*n* = 2,176)^*a*^		
	Cases	Controls	OR^*b*^ (95% CI)	Cases	Controls	OR^*b*^ (95% CI)	Cases	Controls	OR^*b*^ (95% CI)
Total THMs (μg/L × min/day)
< 60.7	550	701	1	363	444	1	185	257	1
60.7–158.7	382	867	0.72 (0.58, 0.89)	244	420	0.76 (0.58, 0.99)	138	447	0.67 (0.47, 0.97)
158.7–336.7	329	920	0.60 (0.48, 0.76)	203	416	0.68 (0.50, 0.92)	126	504	0.54 (0.37, 0.80)
> 336.7	441	781	0.81 (0.63, 1.06)	279	424	0.92 (0.65, 1.29)	162	357	0.71 (0.46, 1.09)
*p-Trend*^*c*^			*0.121*			*0.426*			*0.141*
Chloroform (μg/L × min/day)
< 26.1	550	701	1	361	435	1	188	266	1
26.7–72.5	444	804	0.85 (0.70, 1.03)	284	405	0.90 (0.70, 1.16)	159	399	0.82 (0.59, 1.13)
72.5–143.5	373	861	0.65 (0.53, 0.81)	237	445	0.68 (0.51, 0.89)	136	416	0.68 (0.48, 0.97)
> 143.5	335	903	0.66 (0.52, 0.83)	207	419	0.73 (0.54, 0.99)	128	484	0.60 (0.41, 0.88)
*p-Trend*^*c*^			*< 0.001*			*0.016*			*0.007*
Brominated THMs (μg/L × min/day)
< 16.5	539	712	1	345	455	1	192	257	1
16.5–51.0	339	911	0.66 (0.53, 0.83)	217	431	0.76 (0.57, 1.01)	122	480	0.52 (0.36, 0.74)
51.0–178.0	345	904	0.61 (0.47, 0.80)	220	401	0.78 (0.56, 1.09)	125	503	0.43 (0.28, 0.65)
> 178	479	742	0.90 (0.65, 1.25)	307	417	1.25 (0.83, 1.89)	172	325	0.55 (0.32, 0.95)
*p-Trend*^*c*^			*0.267*			*0.627*			*0.010*
^***a***^Two female cases were excluded from the sex-stratified model because one category in education did not have observations among controls and was dropped from the model. ^***b***^Adjusted for sex, age, geographical area, education, nonsteroidal antiinflammatory drug consumption, smoking, physical activity, and family history of colorectal cancer. The analysis excluded subjects with poor or questionable interviews and with known THM levels for < 70% of the exposure window (from age 18 to 2 years before the interview). ^***c***^Linear trend *p*-value, derived from a likelihood-ratio test comparing a model with the categorical nitrate variable as an ordinal variable (0, 1, 2) with a model that excluded the variable.									

By cancer site, associations for colon and rectal cancer evaluated separately showed similar patterns particularly among men, although slight differences appeared among women (see Table S3). However, a majority of cases (69%) were colon cancers, and the number of cases of rectal cancer were particularly small among women, leading to imprecise estimates.

## Discussion

We estimated long-term exposure to four trihalomethanes in drinking water in a large multicenter case–control study of colorectal cancer, including areas with contrasting THM concentrations and evaluating different routes of exposure. In accord with some experimental evidence, chloroform concentrations were inversely associated with colorectal cancer in men and women. A positive association with colorectal cancer was only identified in the highest exposure category of brominated THM concentrations among men. Similar associations were found for colon and rectal cancer analyzed separately.

Evidence of DBP carcinogenicity in animals differs by chemical, animal species, organ, and administration route. Increased incidence of preneoplastic lesions was found in the colon of rats exposed to chloroform, bromodichloromethane, dibromochloromethane, bromoform, and 3-chloro-4-(dichloromethyl)-5-hydroxy-2(5H)-furanone (MX), individually and in mixtures (McDorman et al. 2003), but was not observed in mice (DeAngelo et al. 2002). Among THMs, bromodichloromethane administered by gavage caused the widest spectrum of neoplasms in rodents, including in the large intestine (Dunnick et al. 1987). However, another study found resistance in the intestinal tract to the effects of MX on preneoplastic or neoplastic development despite the elevated MX genotoxicity observed in the gastrointestinal (GI) tract (Steffensen et al. 1999). Specific evidence on gastrointestinal carcinogenicity has shown that chloroform inhibits chemically induced tumors (Daniel et al. 1989) and nuclear anomalies (Daniel et al. 1991) in rodents exposed to GI carcinogens. More recently, chloroform was identified as the active component of an herbal drug used to treat cancer (Zhang et al. 2014; Yu et al. 2007), and the chloroform fraction of a traditional drug used in Asia has shown antitumor activity (Habib and Karim 2011). Although the mechanisms are not known, a possible explanation would involve selectively destroying the initiated cells (Reddy et al. 1992) through apoptosis and supression of proliferation (Zhang et al. 2014). Finally, the association with chloroform should be cautiously interpreted and remains to be confirmed.

Approximately 11% of the study population had exposures above the maximum contaminant levels for total THMs in Spain (100 μg/L); the maximum concentration observed was 174 μg/L, and most of these high exposures were clustered in the area of Barcelona (97%) followed by Murcia (3%). In Italy, all study subjects were below the regulatory limits (30 μg/L). Specific THMs are not regulated in Italy or in Spain [Diario oficial Boletín Oficial del Estado (BOE) 2003; Gazzetta Ufficiale della Repubblica Italiana (GURI) 2001]. The highest exposure categories defined in previous studies were ≥ 75 μg/L (King et al. 2000), ≥ 46.4 μg/L (Hildesheim et al. 1998) and > 40 μg/L (Young et al. 1987), all of which were included in the range of our study. The case–control study by Bove et al. (2007), which included only men, showed a median chloroform concentration (17.6 μg/L) similar to our median concentration (17 μg/L). Our exposure periods are comparable to those of previous studies, which covered either lifetime (Hildesheim et al. 1998; Young et al. 1987) or 40 years before the interview (King et al. 2000). However, the present study evaluated populations with higher concentrations of brominated THMs than did previous case–control or cohort studies evaluating colorectal cancer. Only one case–control study evaluated associations for brominated THMs (Bove et al. 2007), and it showed positive associations with rectal cancer. Our results are not consistent with those of a cohort study conducted by Doyle et al. (1997), in which the predominant THM was chloroform (geometric mean 46.1 μg/L in surface water, 82% from total THMs), and associations were estimated for this substance. They reported a positive association for colon cancer [Relative risk (RR) = 1.68, 95% CI: 1.11, 2.53], and a null association for rectal cancer (RR = 1.07, 95% CI: 0.60, 1.93) for the highest exposure category (14–287 μg/L). To our knowledge, this is the first epidemiological study reporting a negative association with chloroform. However, King et al. (2000) reported negative associations for some categories of total THM exposure among women, without a clear dose–response. Our findings showing different associations for specific THMs lead us to speculate that the use of total THMs without considering different DBP compositions could at least partly explain the heterogeneous results obtained in previous studies. Finally, the gut microbiota may play a role in colorectal carcinogenesis because chlorinated water has been shown to alter the enteric environment in mice (Sasada et al. 2015). This finding has received recent attention, and evidence is limited.

Whereas negative associations with chloroform were found for both men and women, a positive association with brominated and total THMs was found among men at the high end of the exposure range, where there are few observations and precision is low. Similarly, previous studies have found inconsistent differences between men and women regarding colorectal cancer associated with THM exposure (King et al. 2000). Although associations were minimally modified by introducing covariables in the models (data not shown), residual confounding cannot be ruled out.

Exposure measurement error is a concern in cancer studies given the long exposure windows and limited historical THM measurements. In the present study, we applied different strategies to minimize measurement error for the exposures. Subjects included in the models had exposure estimates covering a major portion of the exposure window (≥ 70%). Because the quality of the interview is related to measurement error (Villanueva et al. 2009), we only analyzed subjects with reliable interviews. Finally, inability to account for THM exposure outside the home may have introduced error in the ingestion estimates for THMs, although most of the total water consumption occurred at home (74%).

We cannot rule out uncontrolled confounding by other water contaminants. However, long-term assessment of exposure to nitrate has been conducted following methods comparable to those used for trihalomethane exposure assessment (Espejo-Herrera et al. 2016). Lifetime average nitrate concentrations in the residence ranged approximately from 3 to 20 mg/L, and Pearson correlation coefficients between long-term nitrate and THM concentrations were 0.03 (range –0.76 to 0.45) for total THMs, –0.41 (range –0.56 to 0.32) for chloroform, and 0.15 (–0.72 to 0.56) for brominated THMs overall (minimum–maximum per area). To explore potential confounding by nitrate, we adjusted our main model additionally for nitrate. The point estimates varied minimally (data not shown), ruling out a major confounding effect of nitrate.

Selection bias might be a concern, particularly in Spain, where response rates were lower than those in Italy. The high response rate in Italy reflects traditionally high compliance of subjects approached for interview in the study’s hospital-based case–control network. In addition, controls were population-based in Spain but hospital-based in Italy, explaining the lower response rates among controls in Spain. However, we assume that the probability of participation is independent from the exposure, and we do not expect an impact on the results from response rates. Study participants in Spain are part of a larger study in which a common pool of controls is shared for different cancer sites (colorectal, breast, prostate, stomach, lymphocytic chronic leukemia), leading to a larger number of controls than of cases and to slightly different age and sex distributions.

Metabolism of DBPs is mediated by enzymes from the GST and CYP families (DeMarini et al. 1997; Pegram et al. 1997; Raucy et al. 1993). In a previous study, we examined interactions between THM exposure and polymorphisms in two of the three genes that modified associations between THMs and bladder cancer (Cantor et al. 2010). We found associations with polymorphisms in *CYP2E1* that were not significant after correction for multiple comparisons. We did not find indications that polymorphisms in *GSTZ* modified the effects of exposure to brominated THMs. Although we evaluated gene–environment interactions in a large study, the analysis was limited by dichotomization of exposure, and our power to detect modest interactions after controlling for multiple comparisons remains limited.

## Conclusions

Associations with colorectal cancer differed for estimated exposures to chloroform versus brominated THMs in residential drinking water in our case–control study. Our findings suggest a protective effect of chloroform, whereas a positive association with brominated THMs was observed among men at the extreme tail of the exposure distribution, where the results may have been driven by a small number of observations. Overall, these results require confirmation.

## Supplemental Material

(924 KB) PDF
