# Supplementary material for: Colorectal Cancer and Long-Term Exposure to Trihalomethanes in Drinking Water: A Multicenter Case–Control Study in Spain and Italy
Source: Environ Health Perspect. 2016 Jul 6;125(1):56–65. doi: 10.1289/EHP155 (PMC5226692; doi:10.1289/EHP155)
Supplement: (924 KB) PDF [file EHP155.s001.acco.pdf]

Environ Health Perspect
DOI: 10.1289/EHP155

Note to readers with disabilities: *EHP* strives to ensure that all journal content is accessible to all readers. However, some figures and Supplemental Material published in *EHP* articles may not conform to 508 standards due to the complexity of the information being presented. If you need assistance accessing journal content, please contact ehp508@niehs.nih.gov. Our staff will work with you to assess and meet your accessibility needs within 3 working days.

Supplemental Material

Colorectal Cancer and Long-Term Exposure to Trihalomethanes in Drinking Water: A Multicenter Case-Control Study in Spain and Italy

Cristina M. Villanueva, Esther Gracia-Lavedan, Cristina Bosetti, Elena Righi, Antonio José Molina, Vicente Martín, Elena Boldo, Nuria Aragonés, Beatriz Perez-Gomez, Marina Pollan, Ines Gomez Acebo, Jone M. Altzibar, Ana Jiménez Zabala, Eva Ardanaz, Rosana Peiró, Adonina Tardón, Maria Dolores Chirlaque, Alessandra Tavani, Jerry Polesel, Diego Serraino, Federica Pisa, Gemma Castaño-Vinyals, Ana Espinosa, Nadia Espejo-Herrera, Margarita Palau, Victor Moreno, Carlo La Vecchia, Gabriella Aggazzotti, Mark J Nieuwenhuijsen, and Manolis Kogevinas

Table of Contents

Table S1. Previous studies evaluating colorectal cancer from individual-based studies (case-control or cohort designs) including incident cases with exposure assessment based on personal information from residential histories

Table S2. Single nucleotide polymorphisms (SNPs) of *CYP2E1* and *GSTZ1* examined in the Spanish population of the study, and interaction p-value for total trihalomethanes (THM), chloroform ($CHCl_3$), and brominated THMs (BrTHM) dichotomized at the percentile 75, that are respectively 60, 20 and 40 μg/l.

Figure S1. Distribution of exposure density of average lifetime THM concentrations in residential tap water (µg/l) among 1837 cases and 3488 controls with exposure estimates ≥70% of the exposure window. Note that scale of x- and y-axes differ by area.

Figure S2. Exposure-response relationship between residential trihalomethane (THM) levels (X axis, in µg/l) and colorectal cancer risk (Y axis, expressed in odds ratios) among 1837 cases and 3454 controls. Odds ratios (95 CI) are adjusted for sex, age, area, education, smoking, physical activity, non-steroidal anti inflammatory drugs and family history of colorectal cancer. Excludes unsatisfactory questionnaires and subjects with THM estimated less than 70% from the exposure window. Tick marks above the x-axes represent observations, and the dashed lines represent the 95% confidence intervals.

Figure S3. Spline of colorectal cancer risk (odds ratio, Y axis) associated with chloroform levels (µg/l, X axis) among men, from generalized additive models adjusted for age, sex, education, smoking, non-steroidal anti inflammatory drugs, smoking, physical activity and family history of colorectal cancer. P-value of gain from the linearity is statistically significant in Barcelona (p-value <0.001), Leon (p-value 0.03), Madrid (p-value 0.04), and Navarra (p-value 0.01). Tick marks above the x-axes represent observations, and the dashed lines represent the 95% confidence intervals. Note that scale of x -axes differ by area.

Figure S4. Spline of colorectal cancer risk (odds ratio, Y axis) associated with total brominated THM levels (µg/l, X axis) among men, from generalized additive models adjusted for age, sex, education, smoking, non-steroidal anti inflammatory drugs, smoking, physical activity and family history of colorectal cancer. P-value of gain from the linearity is statistically significant in Barcelona (p-value <0.001), Cantabria (p-value <0.01), and Navarra (p-value <0.01). Tick marks above the x-axes represent observations, and the dashed lines represent the 95% confidence intervals. Note that scale of x-axes differ by area.

Figure S5. Exposure-response relationship between ingested THM levels (X axis) and colorectal cancer (Y axis, expressed in odds ratios (OR) with 95% confidence intervals (95% CI)) among 2047 cases and 3684 controls. Adjusted for sex, age, area, education, smoking, physical activity, non-steroidal anti inflammatory drugs, and family history of cancer. Excludes unsatisfactory interviews and subjects with less than 70% THM estimated from the exposure window. P-value gain compared to linearity is <0.01 for all models, expect for chloroform in women (p-value=0.32). Tick marks above the x-axes represent observations, and the dashed lines represent the 95% confidence intervals.Note that scale of x-axes differ by area.

Figure S6. Exposure-response relationship between shower-bath THM levels (X axis) and colorectal cancer risk (Y axis, expressed in odds ratios (OR) with 95% confidence intervals (95% CI)) among 1702 cases and 3269 controls. Adjusted for (sex), age, geographical area, education, non steroidal anti inflammatory drugs consumption, smoking, physical activity and family history of colorectal cancer Tick marks above the x-axes represent observations, and the dashed lines represent the 95% confidence intervals. Note that scale of x-axes differ by area.

Table S3. Association between colorectal cancer and average THM concentrations in residential tap water by cancer site among 1273 colon cases, 537 rectal cases, and 3454 controls (27 cases had an unspecified localization).

Table S1. Previous studies evaluating colorectal cancer from individual-based studies (case-control or cohort designs) including incident cases with exposure assessment based on personal information from residential histories

Study design and reference	Country	Recruitment period	Study population	Colon cancer	Rectal cancer	Colorectal cancer	Sex	Exposure index and levels	Exposure window
**Case-control**				OR (95%CI)[a]	OR (95%CI)[a]	OR (95%CI)[a]			
Bove et al. 2007	USA	1978-1986	128 rectal cancer cases<br>253 controls	-	**2.32 (1.22, 4.39)**	-	Men	Bromoform ingested 1.69-15.43 vs. 0.9-0.64 μg/day	Not specified
King et al. 2000	Canada	1992-1994	767 colon cancer cases<br>661 rectal cancer cases<br>1545 controls	**1.87 (1.15, 3.05)**<br>0.92 (0.49, 1.71)	0.98 (0.56, 1.72)<br>0.72 (0.34, 1.53)	Not shown	Men<br>Women	Total trihalomethanes ≥75 vs. ≤24 μg/l	40 years before the study
Hildesheim et al. 1998	USA	1986-1989	560 colon cancer cases<br>537 rectal cancer cases,<br>1983 controls	1.06 (0.7, 1.6)	**1.66 (1.1, 2.6)**	Not shown	All	Total THM ≥46.4 vs. ≤0.7 μg/l	Lifetime
Young et al. 1987	USA	Not specified	347 colon cancer cases<br>611 population controls<br>[and 639 cancer controls]	0.73 (0.44, 1.21)<br>[0.93 (0.55, 1.57)]	-	-	All	>300 vs. <100 mg cumulative total THM exposure	Lifetime
Cragle et al. 1985	USA	1978-1980	200 colon cancer cases<br>407 controls	**3.36 (2.41, 4.61)**	-	-	All	Years living in households receiving chlorinated water, >15 vs. <15 years	1953-1978 (25 years)
**Cohort**				RR (95% CI)[b]	RR (95% CI)[b]	RR (95% CI)[b]			
Koivusalo et al. 1997	Finland	1970-1993	621431 subjects<br>1473 colon cancer cases<br>944 rectal cancer cases	0.83 (0.66, 1.04)<br>0.95 (0.78, 1.85)<br>0.90 (0.77, 1.04)	0.85 (0.66, 1.09)<br>**1.38 (1.03, 1.85)**<br>1.04 (0.86, 1.26)	Not shown	Men<br>Women<br>All	Mutagenicity[c]	1971-1993 (22 years)
				HR (95% CI)[a]	HR (95% CI)[a]	HR (95% CI)[a]			
Doyle et al. 1997	USA	1985-1992	28237 subjects<br>178 colon cancer cases<br>78 rectal cancer cases	**1.68 (1.1, 2.53)**[a]	1.07 (0.60, 1.93)[a]	Not shown	Women	Chloroform, 14-287 μg/l vs. <limit of detection	>10 years before the study

[a] Odds ratio (OR) or hazard ratio (HR) and 95% confidence intervals (CI) correspond to the highest versus lowest exposure categories reported.
[b] Relative risk (RR) and 95% confidence interval (CI) based on continuous variable of exposure and calculated for the mutagenicity level of 3000 net rev/l.
[c] Mutagenicity estimated from an empirical equation relating Ames test results with raw water characteristics and treatment. The risk estimate represents the relative risk of an average exposure in a town using chlorinated surface compared with non chlorinated surface water)

Table S2. Single nucleotide polymorphisms (SNPs) of *CYP2E1* and *GSTZ1* examined in the Spanish population of the study, and nominal interaction p-value for total trihalomethanes (THM), chloroform ($CHCl_3$), and brominated THMs (BrTHM) dichotomized at the percentile 75, that are respectively 60, 20 and 40 μg/l.

gene	SNP	chr	position	A1	A2	HWE	MAF	joint effect nominal *p*-values[a]		
								Total THM	$CHCl_3$	BrTHM
CYP2E1	rs2070673	10	135340567	A	T	0.806	17.5	0.104	0.816	0.233
CYP2E1	rs6413420	10	135340829	T	G	0.748	5.9	0.709	0.613	0.788
CYP2E1	rs943975	10	135342260	C	T	0.911	8.7	0.715	0.969	0.658
CYP2E1	rs915906	10	135343738	C	T	0.359	16.1	0.346	0.221	0.403
CYP2E1	rs8192772	10	135344711	C	T	0.786	7.0	0.169	0.233	0.129
CYP2E1	rs6413421	10	135345811	C	T	1.000	6.0	0.684	0.586	0.730
CYP2E1	rs2070675	10	135346696	T	C	0.189	17.1	**0.029**	0.646	**0.009**
CYP2E1	rs915907	10	135346927	A	C	0.202	14.8	**0.031**	0.392	**0.009**
CYP2E1	rs915908	10	135346959	A	G	0.904	18.1	0.444	0.472	0.413
CYP2E1	rs8192775	10	135348026	A	G	0.365	7.4	0.103	0.383	**0.021**
CYP2E1	rs743535	10	135349367	A	G	0.733	8.5	0.056	0.063	**0.019**
CYP2E1	rs1329149	10	135349801	T	C	0.137	13.0	0.488	0.966	0.883
CYP2E1	rs2515642	10	135352013	C	T	0.916	21.4	0.137	0.436	0.183
CYP2E1	rs2480259	10	135352076	A	G	0.916	21.4	0.124	0.481	0.166
CYP2E1	rs2249694	10	135352153	A	G	0.916	21.4	0.140	0.471	0.186
GSTZ1	rs8177538	14	77787706	G	C	1.000	8.9	0.986	0.922	0.960
GSTZ1	rs3759733	14	77788838	A	G	0.093	41.3	0.837	0.740	0.839
GSTZ1	rs2363643	14	77788908	A	G	0.900	30.4	0.646	0.626	0.700
GSTZ1	rs2287395	14	77791519	G	A	0.119	26.1	0.953	0.868	0.977
GSTZ1	rs7975	14	77793207	A	G	0.294	30.2	0.772	0.632	0.774
GSTZ1	rs7972	14	77793237	A	G	0.423	8.4	0.781	0.750	0.753
GSTZ1	rs1046428	14	77794283	T	C	0.877	22.0	0.294	0.828	0.636

SNP, single nucleotide polymorphism; chr (chromosome); A1, minor allele; A2, wild type allele; HWE, Hardy Weinberg Equilibrium test p-value; MAF, minor allele frequency.

[a] p-value from a likelihood ratio test for the joint effect of the SNP and interaction term, using the dominant model (heterozygous and homozygous variant versus homozygous wild type).

Figure S1. Distribution of exposure density of average lifetime THM concentrations in residential tap water (μg/l) among 1837 cases and 3488 controls with exposure estimates ≥70% of the exposure window. Note that scale of x- and y-axes differ by area.

A) Total trihalomethanes

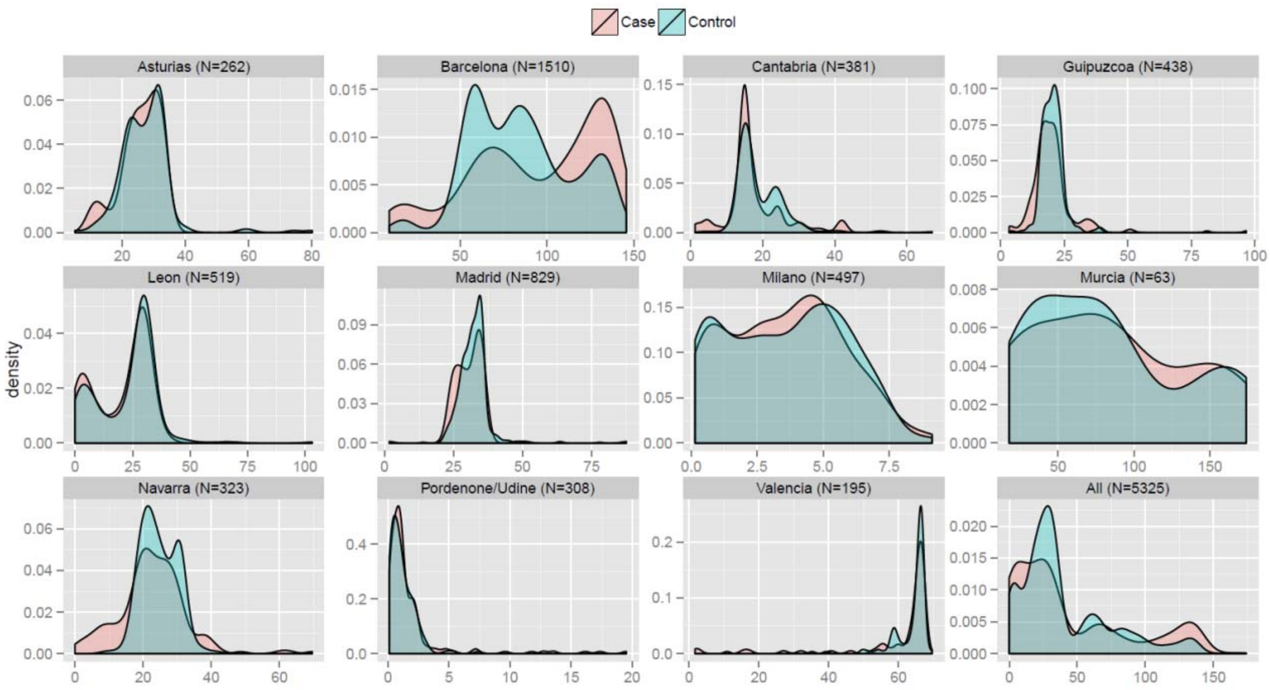

B) Chloroform

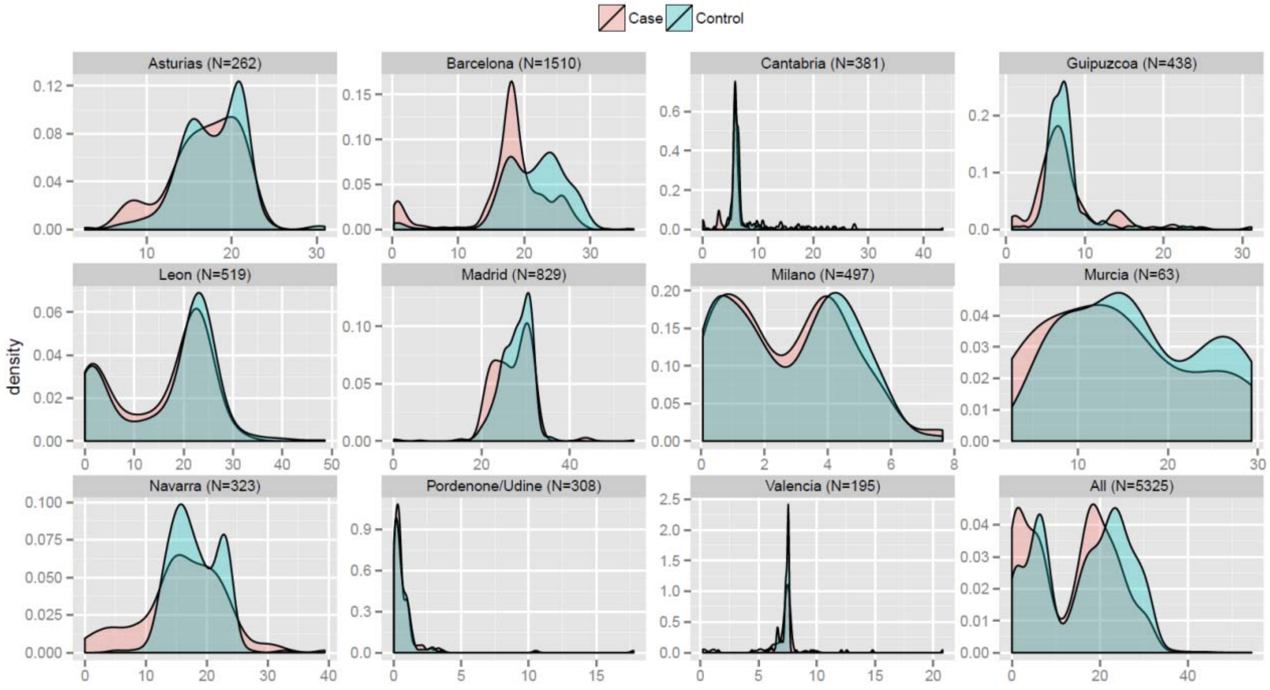

C) Total brominated THMs

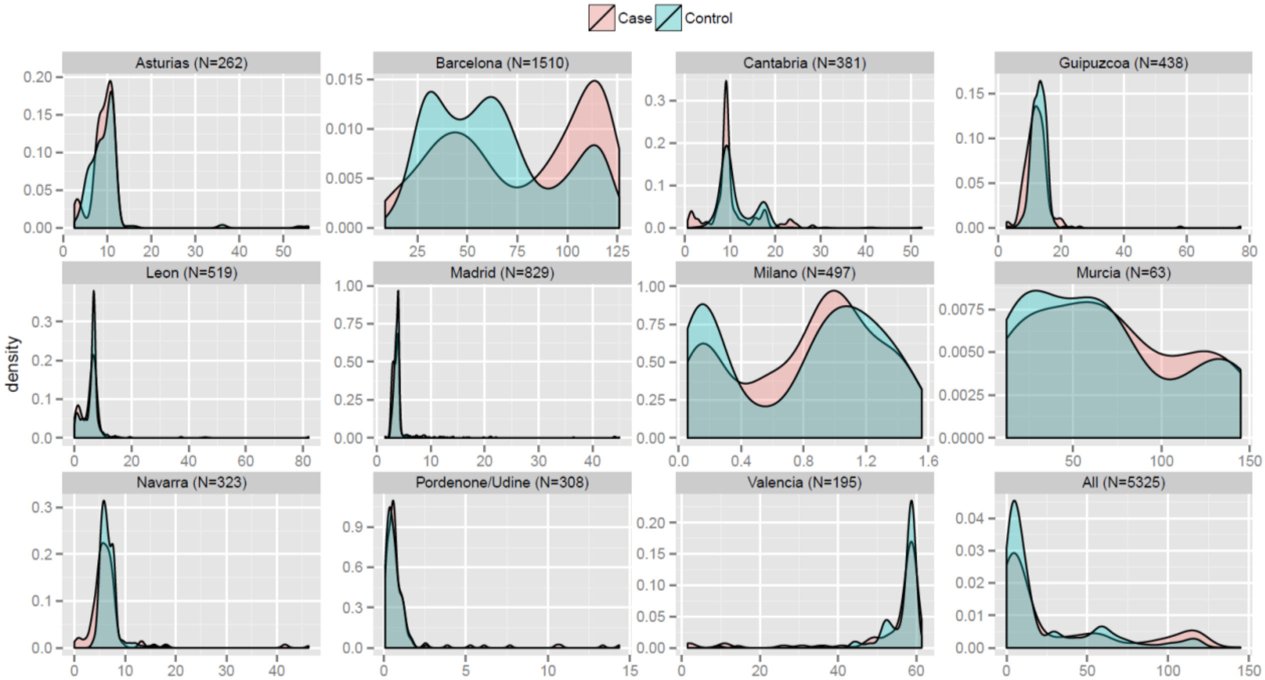

Figure S2. Exposure-response relationship between residential trihalomethane (THM) levels (X axis, in μg/l) and colorectal cancer risk (Y axis, expressed in odds ratios) among 1837 cases and 3454 controls. Odds ratios (95 CI) are adjusted for sex, age, area, education, smoking, physical activity, non-steroidal anti inflammatory drugs and family history of colorectal cancer. Excludes unsatisfactory questionnaires and subjects with THM estimated less than 70% from the exposure window. Tick marks above the x-axes represent observations, and the dashed lines represent the 95% confidence intervals. Note that scale of x -axes differs by chemical.

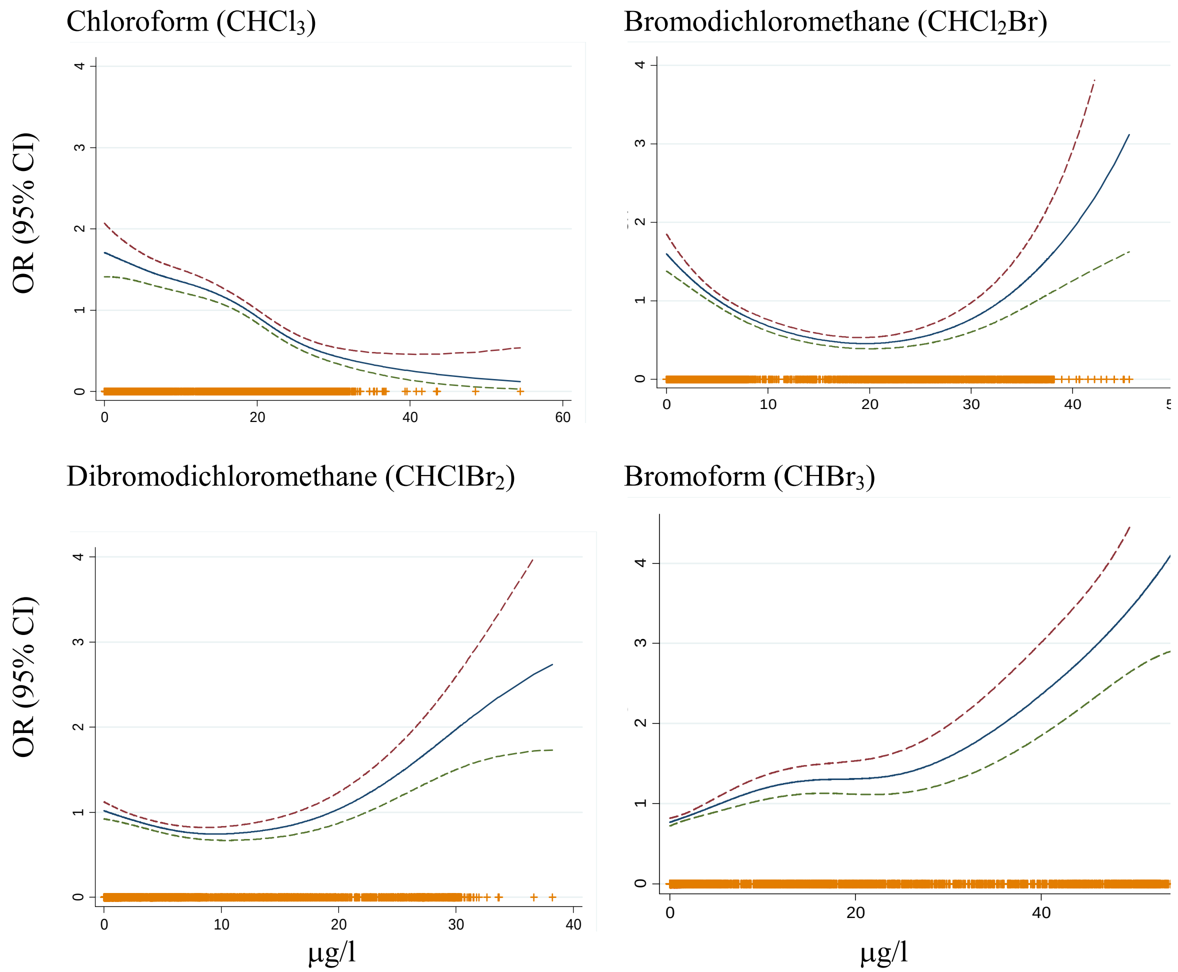

Figure S3. Spline of colorectal cancer risk (odds ratio, Y axis) associated with chloroform levels (μg/l, X axis) among men, from generalized additive models adjusted for age, sex, education, smoking, non-steroidal anti inflammatory drugs, smoking, physical activity and family history of colorectal cancer. P-value of gain from the linearity is statistically significant in Barcelona (p-value <0.001), Leon (p-value 0.03), Madrid (p-value 0.04), and Navarra (p-value 0.01). Tick marks above the x-axes represent observations, and the dashed lines represent the 95% confidence intervals. Note that scale of x -axes differs by area.

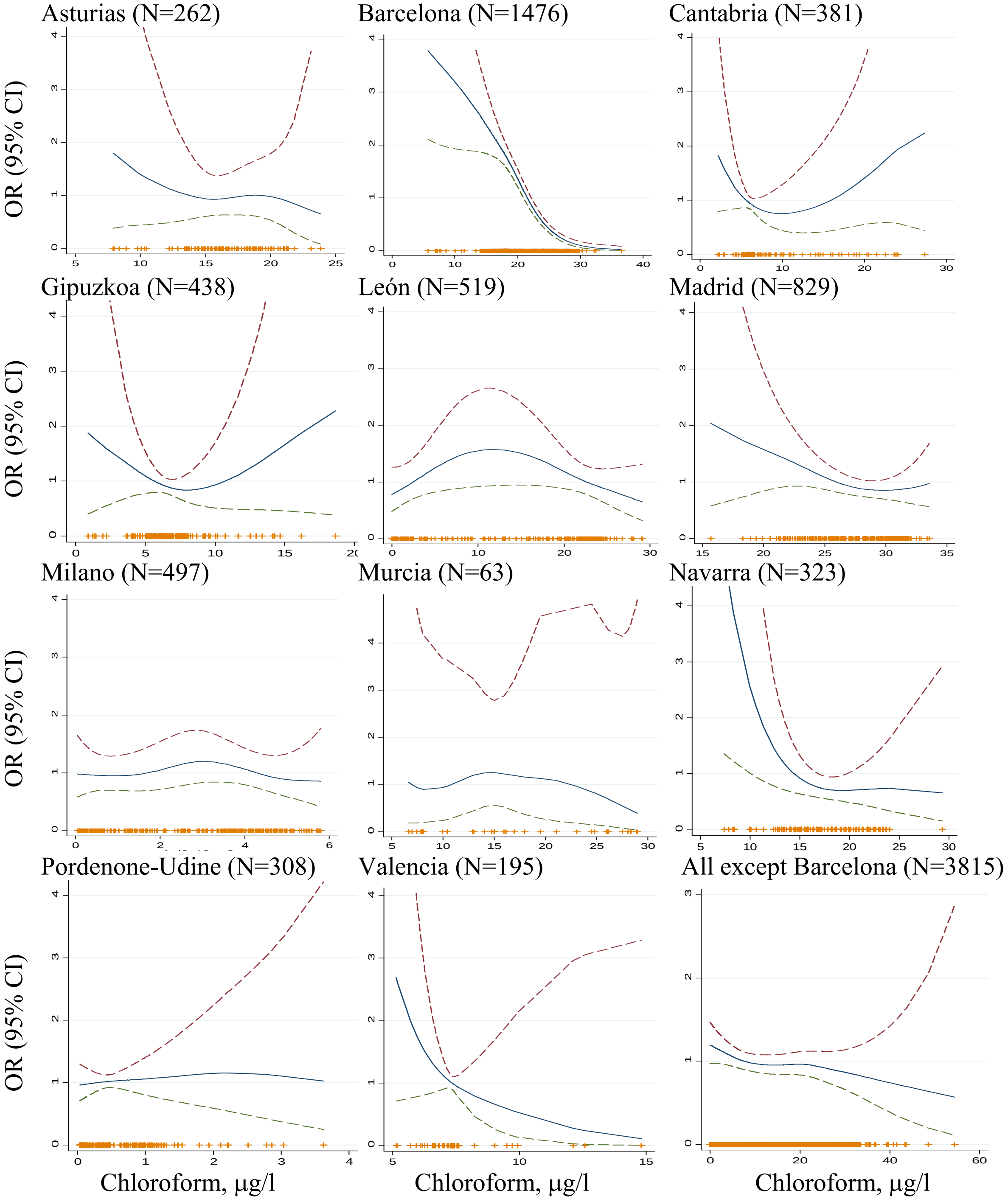

Figure S4. Spline of colorectal cancer risk (odds ratio, Y axis) associated with total brominated THM levels (µg/l, X axis) among men, from generalized additive models adjusted for age, sex, education, smoking, non-steroidal anti inflammatory drugs, smoking, physical activity and family history of colorectal cancer. P-value of gain from the linearity is statistically significant in Barcelona (p-value <0.001), Cantabria (p-value <0.01), and Navarra (p-value <0.01). Tick marks above the x-axes represent observations, and the dashed lines represent the 95% confidence intervals. Note that scale of x-axes differs by area.

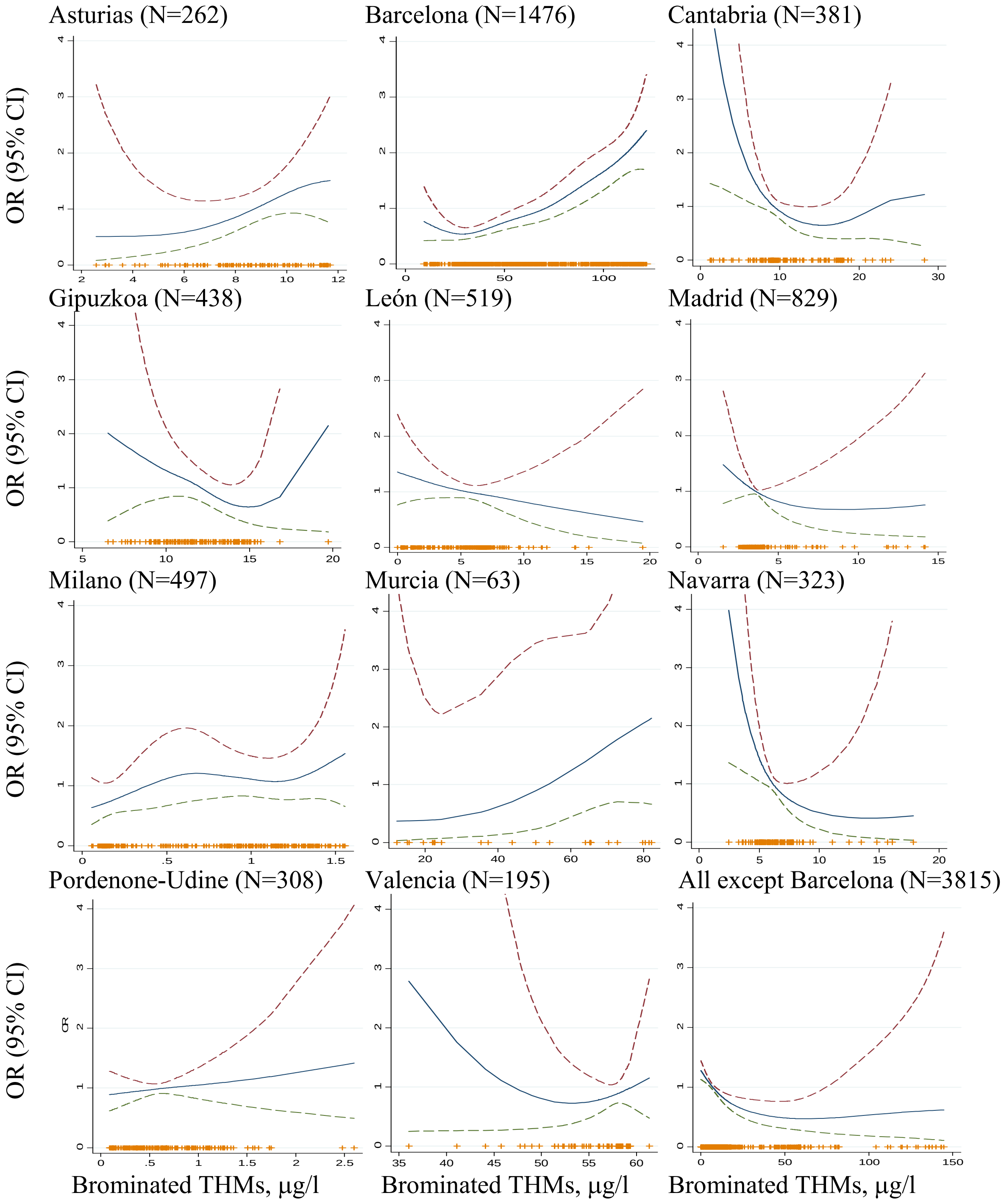

Figure S5. Exposure-response relationship between ingested THM levels (X axis) and colorectal cancer (Y axis, expressed in odds ratios (OR) with 95% confidence intervals (95% CI)) among 2047 cases and 3684 controls. Adjusted for sex, age, area, education, smoking, physical activity, non-steroidal anti inflammatory drugs, and family history of cancer. Excludes unsatisfactory interviews and subjects with less than 70% THM estimated from the exposure window. P-value gain compared to linearity is <0.01 for all models, expect for chloroform in women (p-value=0.32). Tick marks above the x-axes represent observations, and the dashed lines represent the 95% confidence intervals. Note that scale of x-axes differs by area.

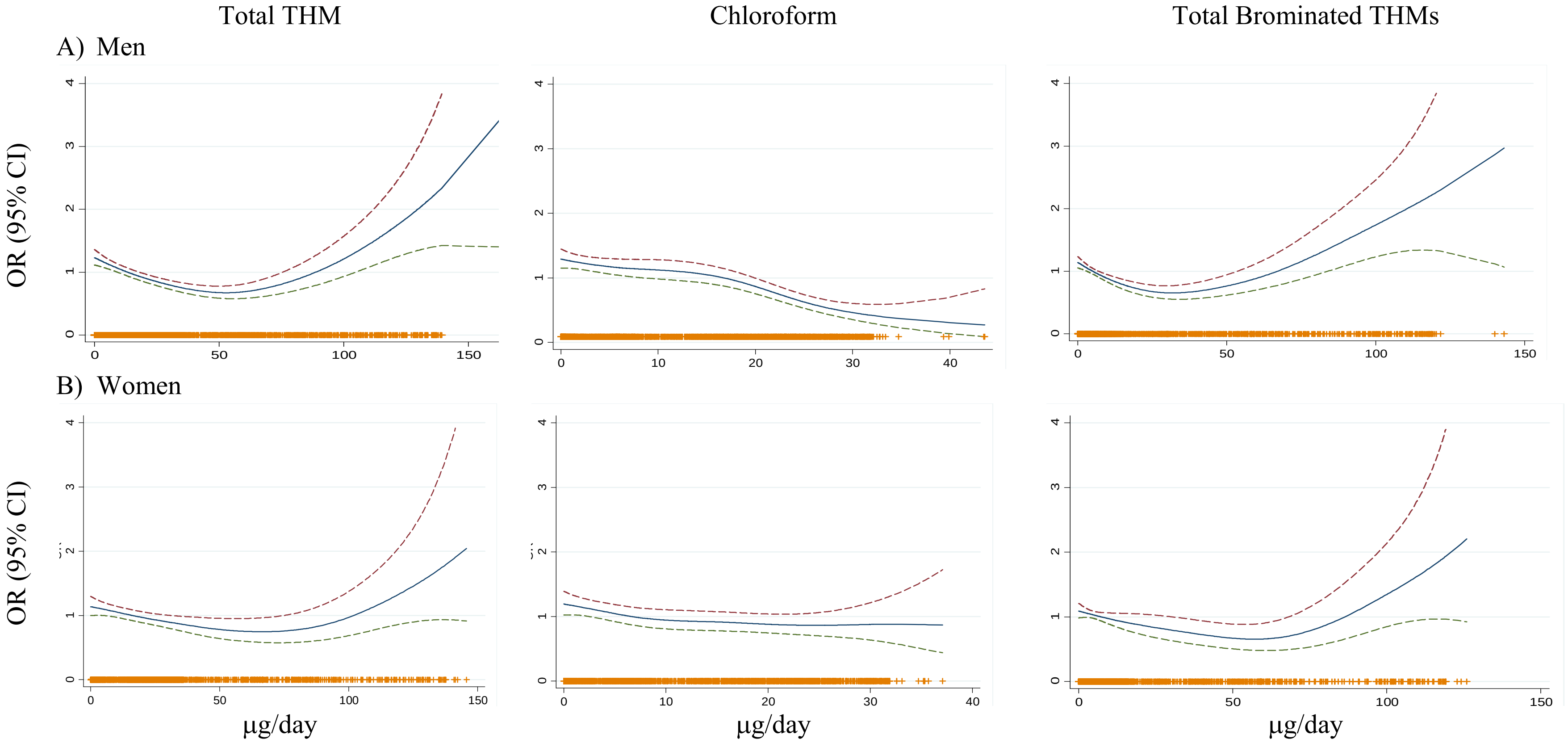

Figure S6. Exposure-response relationship between shower-bath THM levels (X axis) and colorectal cancer risk (Y axis, expressed in odds ratios (OR) with 95% confidence intervals (95% CI)) among 1702 cases and 3269 controls. Adjusted for (sex), age, geographical area, education, non steroidal anti inflammatory drugs consumption, smoking, physical activity and family history of colorectal cancer Tick marks above the x-axes represent observations, and the dashed lines represent the 95% confidence intervals. Note that scale of x-axes differs by area.

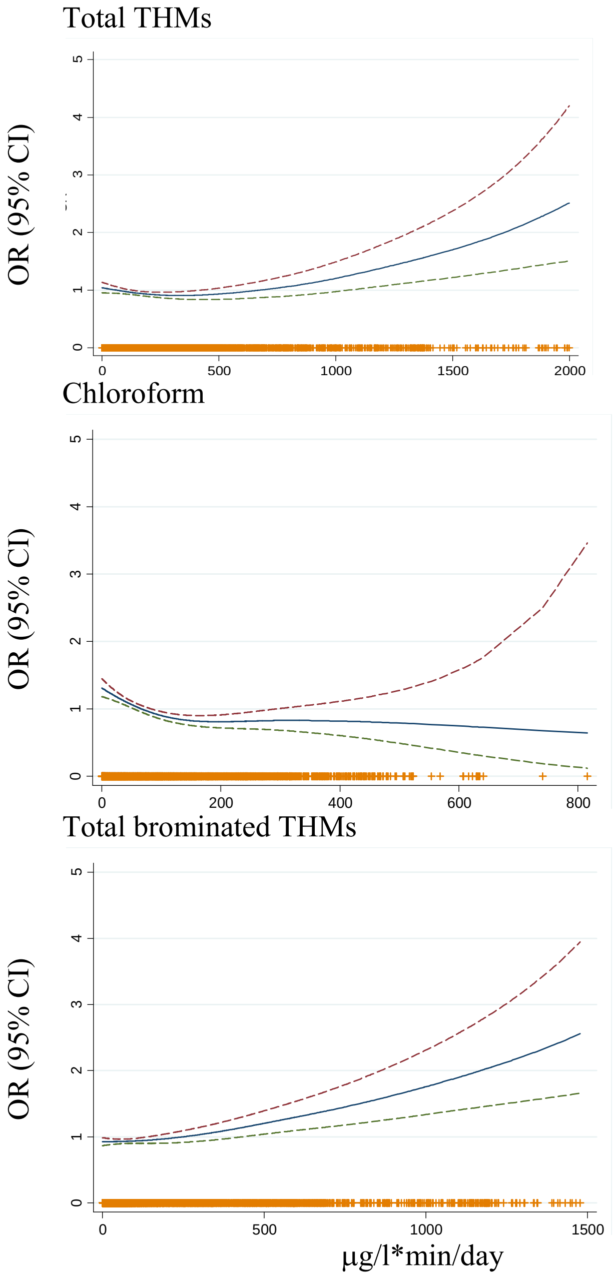

Table S3. Association between colorectal cancer and average THM concentrations in residential tap water by cancer site among 1273 colon cases, 537 rectal cases, and 3454 controls (27 cases had an unspecified localization).

Exposure	**cases**[a]	**contr.**[a]	**OR**[b] **(95% CI)**	**cases**[a]	**contr.**[a]	**OR**[b] **(95% CI)**
			**Colon**			**Rectum**
Chloroform (μg/l), men						
<6	277	446	1	104	445	1
6-17.4	173	404	0.61 (0.44, 0.86)	94	401	0.82 (0.52, 1.27)
17.4-23.4	222	384	0.68 (0.48, 0.98)	108	380	0.75 (0.47, 1.19)
>23.4	120	567	0.24 (0.16, 0.36)	60	567	0.24 (0.14, 0.41)
p-trend[c]			<0.001			<0.001
Chloroform (μg/l), women						
<6	147	298	1	46	298	1
6-17.4	120	491	0.63 (0.42, 0.95)	46	491	1.08 (0.52, 2.26)
17.4-23.4	127	414	0.52 (0.34, 0.81)	53	413	1.21 (0.56, 2.62)
>23.4	85	450	0.40 (0.24, 0.67)	26	450	0.64 (0.26, 1.56)
p-trend[c]			0.001			0.336
Total brominated THM (μg/l), men						
<3.7	247	483	1	93	481	1
3.7-9	154	419	0.73 (0.51, 1.05)	81	416	0.74 (0.45, 1.21)
9-41.8	173	479	0.67 (0.39, 1.14)	65	478	0.34 (0.16, 0.71)
>41.8	218	420	1.42 (0.76, 2.65)	127	418	1.44 (0.60, 3.46)
p-trend[c]			0.093			0.037
Total brominated THM (μg/l), women						
<3.7	133	309	1	51	309	1
3.7-9	119	524	0.79 (0.51, 1.25)	30	524	0.68 (0.33, 1.40)
9-41.8	89	484	0.35 (0.17, 0.72)	37	484	1.12 (0.39, 3.27)
>41.8	138	336	0.31 (0.13, 0.72)	53	335	1.21 (0.35, 4.25)
p-trend[c]			0.017			0.894

[a] Numbers do not always add 1273 colon cases, 537 rectal cases, and 3454 controls due to observations dropped from the analyses because of insufficient observations in some covariable categories.

[b] Odds ratio (OR) and 95% confidence interval (CI) adjusted for sex, age, area, education, smoking, physical activity, non-steroidal anti inflammatory drugs, family history of colorectal cancer. Excludes unsatisfactory questionnaires and subjects with THM estimated less than 70% from the exposure window.

[c] Linear trend p-value, derived from a likelihood ratio test comparing a model with the categorical nitrate variable as an ordinal variable (0, 1, 2), with a model that excluded the variable.

REFERENCES

Bove GE, Jr., Rogerson PA, Vena JE. 2007. Case control study of the geographic variability of exposure to disinfectant byproducts and risk for rectal cancer. Int J Health Geogr 6:18.

Cragle,D.L., Shy,C.M., Struba,R.J. and Siff,E.J.: 1985, A case-control study of colon cancer and water chlorination in North Carolina. In: Jolley R.L., Bull R.J., Davis W.P., Katz S., Roberts M.H.Jr. and Jakobs V.A. (eds.), Water Chlorination: chemistry, environmental impact and health effectsLewis Publishers, Inc., Chelsea, MI (USA), pp. 153-160.

Doyle TJ, Zheng W, Cerhan JR, Hong CP, Sellers TA, Kushi LH et al. 1997. The association of drinking water source and chlorination by- products with cancer incidence among postmenopausal women in Iowa: a prospective cohort study. Am J Public Health 87:1168-1176.

Hildesheim ME, Cantor KP, Lynch CF, Dosemeci M, Lubin J, Alavanja M et al. 1998. Drinking water source and chlorination byproducts. II. Risk of colon and rectal cancers. Epidemiology 9:29-35.

King WD, Marrett LD, Woolcott CG. 2000. Case-control study of colon and rectal cancers and chlorination by-products in treated water. Cancer Epidemiol Biomarkers Prev 9:813-818.

Koivusalo M, Pukkala E, Vartiainen T, Jaakkola JJ, Hakulinen T. 1997. Drinking water chlorination and cancer-a historical cohort study in Finland. Cancer Causes Control 8:192-200.

Young TB, Wolf DA, Kanarek MS. 1987. Case-control study of colon cancer and drinking water trihalomethanes in Wisconsin. Int J Epidemiol 16:190-197.
